# Endocan Promotes Pro-Tumorigenic Signaling in Lung Cancer Cells: Modulation of Cell Proliferation, Migration and lncRNAs H19 and HULC Expression

**DOI:** 10.3390/ijms24098178

**Published:** 2023-05-03

**Authors:** Federica Aliquò, Aurelio Minuti, Angela Avenoso, Giuseppe Mandraffino, Giuseppe Maurizio Campo, Salvatore Campo, Angela D‘Ascola, Michele Scuruchi

**Affiliations:** 1 Department of Biomedical and Dental Sciences and Morphofunctional Images, University of Messina, Via C. Valeria, 98125 Messina, Italy; 2 Department of Clinical and Experimental Medicine, University of Messina, Via C. Valeria, 98125 Messina, Italy

**Keywords:** endocan, proteoglycans, tumors, NSCLC, angiogenesis, long non-coding RNAs

## Abstract

Endocan is a circulating proteoglycan secreted by several cell lines and identified as a potential biomarker of inflammation and angiogenesis. Endocan-increased expression has been found in a broad spectrum of human tumors, including lung cancer, and is associated with a poor prognosis. To elucidate the possible mechanism, this study aimed to investigate the role of endocan in non-small-cell lung carcinoma (NSCLC) using an in vitro model of cultured cells. Endocan expression was knocked down by using a specific small interfering RNA. The effects of endocan knockdown have been evaluated on VEGF-A, VEGFR-2, HIF-1α, the long non-coding RNAs H19 and HULC expression, and AKT and ERK 1/2 degree of activation. Cell migration and proliferation have been studied as well. VEGF-A, VEGFR-2, HIF-1α, and the long non-coding RNAs H19 and HULC expression were significantly affected by endocan knockdown. These effects correlated with a reduction of cell migration and proliferation and of AKT and ERK 1/2 activation. Our findings suggest that endocan promotes a more aggressive cancer cell phenotype in NSCLC.

## 1. Introduction

Lung cancer is one of the most common causes of cancer-related death worldwide, with an estimated 2.20 million new cases and 1.79 million deaths yearly [1]. Recently, the discovery of predictive biomarkers has led to the development of innovative therapeutic approaches through the use of targeted therapy and immunotherapy [2]. The term lung cancer refers to several tumoral conditions consisting of different tumor subtypes, each of which carries a different molecular pattern and pathologic profile. Among them, non-small cell lung cancer (NSCLC) is the main malignant epithelial tumor of the lung and includes three histological subtypes: lung adenocarcinoma (ADC), lung squamous cell carcinoma (SqCC), and large cell lung carcinoma (LCC); although the etiology of such subtypes remains not well established, it is believed that they arise from different cells of origin [3].

In NSCLC, the extracellular matrix (ECM) plays an extremely relevant role, providing histoarchitectural support and anchoring the cells. Furthermore, ECM molecules regulate cell survival, proliferation, differentiation, and motility, mediating important signaling pathways, and the impaired expression of such molecules promotes invasive carcinoma with an important impact on clinical outcomes [4,5].

ECM is a complex network of macromolecules and signaling factors that interact to maintain the structural and functional integrity of tissues [6]. Proteoglycans (PGs) are the main components of the ECM. They consist of a central core protein onto which one or more glycosaminoglycan (GAG) chains are covalently linked, including chondroitin sulphate (CS), dermatan sulphate (DS), keratan sulphate (KS), heparin (HP), and heparan sulphate (HS). PGs are membrane-bound, intracellular, or secreted molecules embedded in the ECM. The impaired regulation of such compounds has been reported in human cancers where, through their protein core and/or GAG chains, they interact with a wide array of molecules participating in several pathophysiological steps of tumor formation and progression [6,7,8,9]. In this setting, it has been shown that by binding extracellular ligands, cell surface receptors, and other extracellular matrix molecules, PGs engage cell signaling pathways involved in cell proliferation, cell adhesion, and cell motility [7].

Endocan, also known as endothelial specific molecule-1 (ESM-1), is a circulating PG secreted by several cell lines and involved in a plethora of pathological conditions, such as cancer and inflammatory diseases [10,11]. This PG was first cloned from the human umbilical vein endothelial cells (HUVECs) cDNA library in 1996 by Lassalle et al. as a constitutively secreted molecule [12]. The presence of a dermatan sulphate (DS) chain linked to the protein core identified ESM-1 as a dermatan sulphate proteoglycan (DSPGS) under the name of endocan [10]. Data have shown that the expression of such PG may be modulated by several pro-inflammatory cytokines and pro-angiogenic growth factors. For instance, vascular endothelial growth factor A (VEGF-A), fibroblast growth factor (FGF-2), transforming growth factor-β1 (TGF-β1), and interleukin-1β (IL-1β) have been shown to strongly upregulate endocan expression [13,14]. In contrast, interferon-γ (IFN-γ) inhibits its expression [12].

Multiple signaling pathways are involved in regulating endocan expression, such as the PKC/NF-kB and the PI3K/AKT pathways [15]. Furthermore, in hypoxic conditions, it has been shown that activated hypoxia-inducible factor 1 alpha (HIF-1α) stimulates vascular endothelial growth factor (VEGF) expression, which in turn mediates endocan expression in a VEGF/VEGFR-2 dependent manner [16,17]. Increased serum endocan levels have been correlated with tumor aggressiveness and vascularization [18]. In light of this evidence, endocan could be considered a potential cancer biomarker [19].

Endocan is overexpressed in many human tumors, including lung cancer [20]. In this setting, in a gene profiling study in tissues from 23 patients, among 42 genes associated with a high risk for cancer death, endocan has been identified as one of the most significant molecular signatures associated with a worse prognosis [20]. Furthermore, in NSCLC, high endocan levels have been positively associated with the development of distant metastases and, thus, a poor prognosis [21,22].

Long non-coding RNAs (lncRNAs) represent a novel class of functional molecules that are critically involved in cancer biology, including NSCLC.

LncRNAs are a class of non-coding RNAs longer than 200 nucleotides that can affect gene expression by interacting with DNA, RNA, and proteins and regulating RNA splicing, stability, and translation [23].

Several studies have reported that lncRNAs are aberrantly expressed in cancer, and their deregulation generally contributes to tumor progression by promoting proliferation, invasion, and metastasis of tumor cells. In this setting, these non-coding RNAs act as oncogenic or tumor suppressors [24,25,26,27], thereby they may be considered potential therapeutic targets and biomarkers for diagnosis, prognosis, and/or treatment, owing to their characteristics of high efficiency, high tissue specificity, and stability [28,29]. Among them, the lncRNA H19 is involved in developing multiple tumors, including lung cancer [30]. In this setting, elevated plasma levels of H19 were found in NSCLC patients, and it has been proposed as a promising biomarker for diagnosing this type of cancer [31]. 

It was shown that higher levels of H19 were associated with a poorer prognosis in ovarian cancer (OC). Its knockdown by small interfering RNA inhibited OC cell migration and invasion both in vitro and in vivo [32]. In bladder cancer, upregulated H19 promoted cell migration and metastasis by modulating the Wnt/β catenin pathway [33]. 

The same effects have been observed in glioma, where it has been found that such lncRNA promotes cell proliferation, migration, and angiogenesis by regulating the Wnt5a/β-catenin pathway and targeting miR-342 [34] and enhancing HIF-1α/VEGF signaling [35]. 

Similar functions have been reported for the highly upregulated liver cancer (HULC) lncRNA, whose expression has been correlated with increased endocan and VEGF expression and enhanced angiogenesis and tumor progression [36]. In osteosarcoma, HULC overexpression enhanced cell proliferation, migration, and invasion by blocking PTEN and increasing the activity of the AKT-PI3K-mTOR pathway [37]. In addition, HULC promotes mesenchymal stem cells (MSCs) migration and invasion by enhancing the expression of vimentin, N-cadherin, and MMP2 [38]. 

As known, the PI3K/AKT and MAPK/ERK signaling pathways are dysregulated in a broad spectrum of human cancers, including lung cancer [39,40,41]. In this setting, their aberrant activation drives carcinogenesis by regulating cell survival, apoptosis, growth, proliferation, and migration [42]. Akt is the central node of the PI3K/AKT pathway; this molecule promotes tumor cell survival by inactivating pro-apoptotic proteins, including procaspase-9 and BCL-2 [43]. Furthermore, Akt promotes cell proliferation by phosphorylating glycogen synthase kinase 3β (GSK3β), thus preventing cyclin D1 degradation [44].

As a member of the mitogen-activated protein kinase (MAPK) family, ERK1/2 regulates key biological functions [45]. Activated ERK 1/2 migrates into the nucleus and phosphorylates numerous transcription factors, such as c-Fos, c-Myc, and c-Jun, promoting cell growth, survival, and proliferation [46]. 

In light of the evidence, in the present study, we aimed to investigate the effects of endocan knockdown on VEGF-A, VEGFR-2, HIF-1α, the long non-coding RNAs H19, and HULC expression in A549 cells as a model of NSCLC. Furthermore, we also studied if endocan silencing affected AKT and ERK 1/2 activation as well as cell migration and proliferation. 

## 2. Results

### 2.1. Evaluation of Endocan Expression in A549 Cells

**Figure 1:**
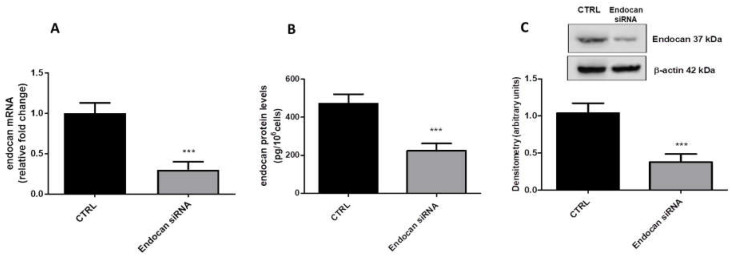
Endocan mRNA expression (**A**) and related protein levels (**B**,**C**) in endocan knockdown and control A549 cells. Data are the mean ± SD of five experiments, mRNA levels are expressed as relative fold change; protein levels are expressed as pg/10^6^ cells (ELISA assay) and as arbitrary units (western blot). *** *p* < 0.001 vs. CTRL.

Endocan mRNA and related protein expression have been reported to be overexpressed in several types of cancer, including NSCLC [47]. As shown, also in our model, endocan was expressed, as confirmed by qPCR, ELISA assay, and western blot data (Figure 1A–C). 

To obtain endocan knockdown, we treated A549 cells with a specific small interfering RNA against endocan mRNA. As reported, the treatment with the endocan siRNA was able to significantly reduce the expression of such PG in terms of both mRNA (panel A) and protein levels (panels B and C). To note, endocan knockdown reduced both intracellular (panel C) and secreted protein levels (panel B). These results indicated a high efficiency of endocan knockdown in our model.

### 2.2. Endocan Silencing Affects VEGF-A, VEGFR-2, HIF-1α, and Long Non-Coding RNAs H19 and HULC Expression in A549 Cells 

**Figure 2:**
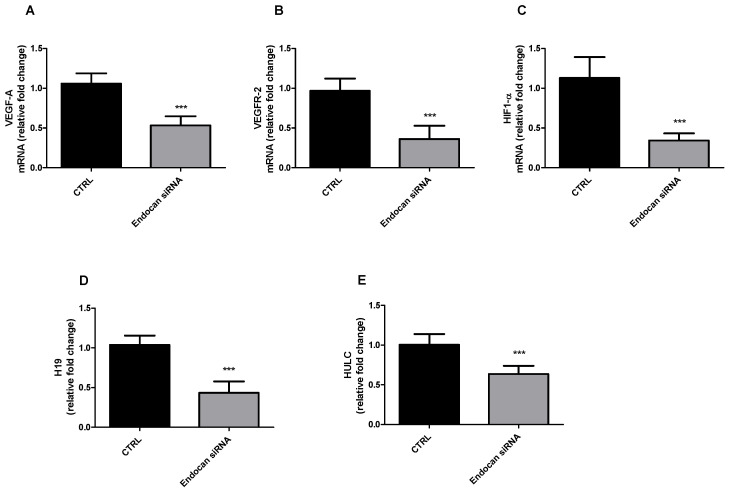
VEGF-A (**A**), VEGFR-2 (**B**), HIF-1α (**C**), H19 (**D**), and HULC (**E**) expression assessed by q-PCR in endocan knockdown and control A549 cells. Data are the mean ± SD of five experiments and are expressed as relative fold change. *** *p* < 0.001 vs. CTRL.

Since it has been shown that endocan supports tumor progression and promotes angiogenesis within the tumor by modulating VEGF-A and HIF-1α expression and signaling [47], we studied the effect of endocan knockdown on *VEGF-A, VEGFR-2, HIF-1α* mRNA expression. qPCR results reported in Figure 2A–C show that VEGF-A, VEGFR-2, and HIF-1α mRNA expression was significantly reduced in cells where endocan was knocked down if compared to control cells. These results, in line with previous evidence, demonstrate that endocan mediates the expression of such angiogenic factors in A549 cells as well. 

LncRNAs play key roles in regulating tumorigenesis and tumor progression by modulating gene expression through transcriptional regulation, epigenetic regulation of chromatin modification, and post-transcriptional regulation of target genes [48]. It has been reported that H19 and HULC are involved in the epigenetic mechanisms that regulate NSCLC development [49,50,51]. Therefore, we aimed to evaluate the effects of endocan knockdown on the expression of lncRNAs H19 and HULC.

As reported in Figure 2D,E, we found a significant reduction of H19 (panel D) and HULC (panel E) expression in cells treated with the specific endocan siRNA compared to control cells. Our results show that endocan can control H19 and HULC expression in A549 cells, suggesting a new and interesting role for such PG in NSCLC pathobiology.

### 2.3. Endocan Silencing Inhibits Cell Proliferation and Migration in A549 Cells

**Figure 3:**
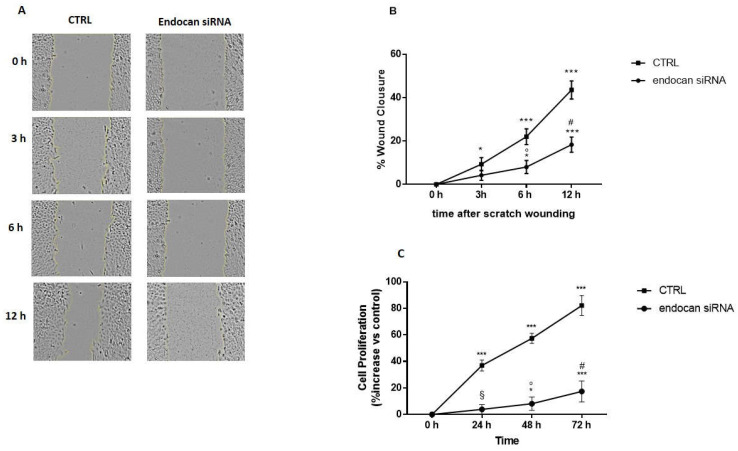
Effects of endocan siRNA on A549 cell migration (**A**,**B**). Representative wounds immediately after scratching and after 3, 6, and 12 h of healing were recorded with a phase-contrast microscope (**A**). The wound area was measured at 0, 3, 6, and 12 h after scratching to determine the area over which healing occurred, and results are expressed as a percentage of wound closure (**B**). Error bars represent the mean ± SD of almost three independent experiments. *** *p* < 0.001 and * *p*< 0.05 vs. CTRL at 0 h; ° *p* < 0.05 vs. CTRL at 6 h: ^#^ *p* < 0.01 vs. CTRL at 12 h. Effect of endocan siRNA on A549 cell proliferation (**C**). The data are expressed as percentage of increase vs. control. Error bars represent the mean ± SD of almost three independent experiments. *** *p* < 0.001, and * *p* < 0.05 vs. CTRL at 0 h; ^§^ *p* < 0.001 vs. CTRL at 24 h; ° *p* < 0.001 vs. 48 h CTRL at 48 h, ^#^*p* < 0.001 vs. CTRL at 72 h.

Tumor progression is driven by the uncontrolled proliferation, migration, and invasion of cancer cells that migrate from their primary sites into new distant organs and tissues [52]. To study the involvement of endocan in NSCLC progression, we first measured cell migration in control and endocan knockdown A549 cells by performing a scratch migration assay. In Figure 3, representative images of the wound (panel A) and the migration rate (panel B) at 0 h, 3 h, 6 h, and 12 h are reported. As shown, the capacity to fill the wound was severely inhibited in A549 cells, where endocan expression was blocked by transfection with the endocan siRNA (panel A). These results were confirmed by measuring the migration rate (panel B); in fact, the percentage of wound closure values in control cells showed a 40% reduction of the open wound area after 12 h. These results clearly show that A549 cells transfected with the specific endocan siRNA are characterized by a reduced migratory capacity compared to control cells. 

Next, we measured cell proliferation by performing an MTT assay in both control and endocan knockdown A549 cells. The growth was measured at 0 h, 24 h, 48 h, and 72 h. As reported in Figure 3C, the proliferation rate was significantly reduced, within the all-time intervals considered, in both endocan knockdown A549 cells and control cells. Taken together, these results suggest an important role for endocan in promoting NSCLC cell migration and proliferation. 

To determine whether cells underwent apoptosis, we evaluated caspase-3 activity using a Caspase-3/CPP32 Colorimetric Protease Assay Kit (Thermo Fisher Scientific, Milano, Italy). As reported in Supplementary data, the knockdown endocan did not induce apoptosis in A549 cells (Figure S1).

### 2.4. Endocan Silencing Decreased AKT and ERK1/2 Activation

The PI3K/AKT and MAPK/ERK signaling pathways are aberrantly activated in a wide spectrum of human tumors, including NSCLC, where they promote tumor cell migration and proliferation [53].

**Figure 4:**
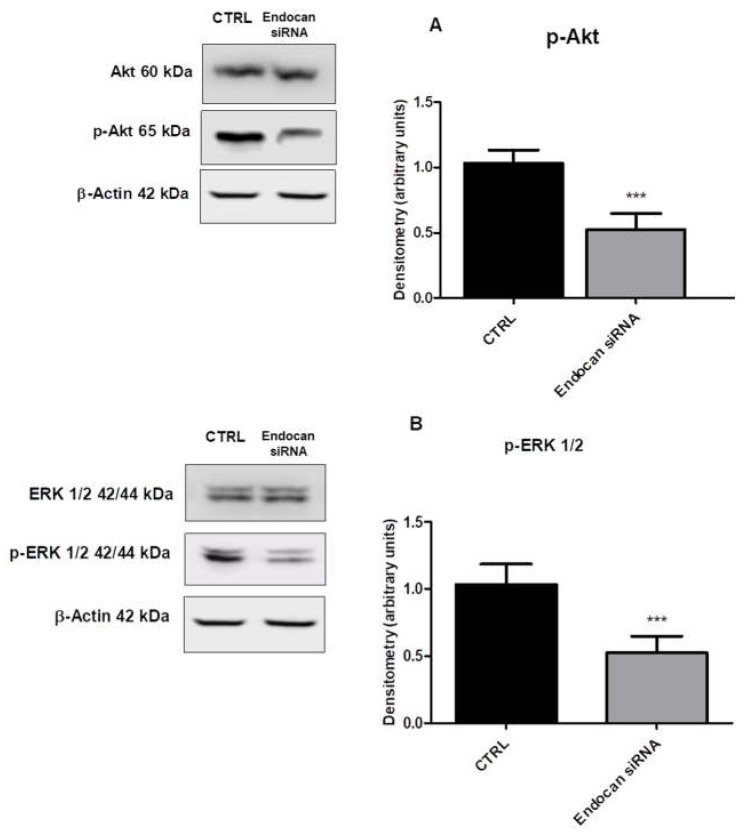
p-AKT, AKT, p-ERK 1/2, and ERK 1/2 protein levels in control and endocan knockdown A549 cells. (**B**) Average ERK1/2 phosphorylation is quantified as ratios between p-ERK1/2 and ERK1/2. (**A**)Average AKT phosphorylation is quantified as ratios between p-AKT and AKT. Data are the mean ± SD of five experiments and are expressed as both western blot analysis and densitometric evaluation. *** *p* < 0.001 vs. CTRL.

Based on our obtained data regarding endocan effects on cell migration and proliferation, we assessed the downstream signaling by evaluating the degree of AKT and ERK1/2 activation in A549 cells endocan knocked down compared to control cells. In Figure 4, western blot data with the densitometric evaluation of p-AKT/AKT (panel A) and p-ERK1/2/ERK1/2 (panel B) are reported. As shown, the degree of AKT and ERK-1/2 phosphorylation was significantly decreased in cells where endocan expression was inhibited by endocan siRNA compared to control cells. Together, these data suggest that endocan promotes cell migration and proliferation, modulating these downstream pathways in NSCLC. 

## 3. Discussion

Endocan is known to be involved in a broad spectrum of malignant tumors, inflammatory diseases, and vascular disorders [54,55]. In the context of the pathophysiology of the lung, elevated endocan levels have been found in patients with lung cancer and are associated with poor clinical outcomes [21]. Endocan expression and functions are strictly related to VEGF signaling; in this setting, it has been shown that VEGF stimulates endocan secretion, which in turn enhances its pathway, promoting the interaction of such pro-angiogenic factor with VEGFR-2. Furthermore, endocan can modulate the expression of angiogenesis-related genes, including VEGF-A and VEGFR-2 [47,56].

The VEGF family includes different VEGF isoforms; among these, VEGF-A is a key factor in normal and tumor-associated angiogenesis and exerts its physiological functions by engaging three types of receptors: VEGFR-1, VEGFR-2, and VEGFR-3 [57]. In particular, VEGFR-2 is critically involved in tumor angiogenesis, and its activation promotes neighboring vessel formation for the sustainability of cancer proliferation, migration, metastasis, and survival [58].

A hypoxic environment is an essential and common feature of solid tumors; in this setting, the HIF-1α activity promotes tumor cells’ adaptation to the low oxygen tension [59]. In particular, activated HIF-1α is responsible for the transcriptional activation of several genes, including VEGF, orchestrating both normal and pathological angiogenesis [60]. In addition, it has been shown that HIF-1α-mediated VEGF expression correlates with the increased expression of endocan [47]. 

This evidence led to the hypothesis that endocan could influence the expression of these angiogenesis-associated genes in NSCLC as well.

In the present study, by evaluating VEGF-A, VEGFR-2, and HIF-1α, we found that in cells where endocan was silenced by the specific siRNA, the expression of such factors was significantly reduced. These results, in line with previous evidence [47,56], lead to the hypothesis that endocan, once released, upregulates the expression of such pro-angiogenic factors and promotes NSCLC growth in an autocrine and paracrine manner. Therefore, the inhibition of endocan expression may inhibit this network.

LncRNAs are key players in different cellular functions, including regulating cell proliferation, invasion, and metastasis, thus acting as oncogenes or tumor suppressors [61].

In this context, the dynamic interplay between lncRNAs and ECM components plays a critical role in the pathobiology of human diseases, including lung cancer, where lncRNAs have been proposed as potential cancer biomarkers [62]. Among these, the altered regulation of lncRNAs H19 and HULC transcription has been reported in many types of tumors, and their aberrant expression triggers the activation of genes deeply involved in cell survival, proliferation, and angiogenesis [63,64]. 

Based on these previous reports, we evaluated the effects of endocan knockdown on H19 and HULC expression, and we found that the expression of these lncRNAs was significantly reduced in cells where endocan expression was blocked compared to control cells. 

It has been shown that in lung cancer, higher expression of H19 is positively correlated with poor prognosis, and its knockdown significantly reduces tumor cell proliferation, suggesting that H19 plays a pivotal role in NSCLC progression [49,50]. Furthermore, Liu et al. showed that overexpression of HULC, another cancer-related lncRNA, enhances sphingosine kinase 1 (SPHK1) expression and AKT phosphorylation, thus promoting tumor cell proliferation. Furthermore, they showed that treatment with a specific SPHK1 inhibitor reduced these HULC modulatory effects [51]. 

Our results further enforce these data and clearly show that endocan affects H19 and HULC expression, suggesting a possible regulatory mechanism of endocan on these lncRNAs in NSCLC. 

The PI3K/AKT and MAPK/ERK pathway’s aberrant activation is a hallmark of tumor progression and resistance to cancer therapies [53,65,66]. Increased AKT activity has been reported in patients with NSCLC, and it has been associated with a poor prognosis [67]. Furthermore, it has been reported that endocan promotes triple-negative breast cancer cell proliferation in an AKT-dependent manner [68]. 

In light of these findings, we also evaluated the effects of endocan knockdown on AKT activation in our experimental model. As shown, we found that the degree of AKT phosphorylation was significantly reduced in cells where the expression of such PG was inhibited. 

ERK 1/2 is a downstream effector of VEGF signaling, and it has been shown that endocan modulates its expression by inducing ERK 1/2 phosphorylation [56]. In the cancer context, Jin and colleagues have shown that endocan overexpression correlated with increased breast cancer cell proliferation and migration due to enhanced ERK 1/2 activity; they also found that the degree of ERK1/2 activation was strongly reduced in cells where the expression of endocan was inhibited. According to these modulatory effects, they concluded that endocan plays a crucial role in radiotherapy-resistant breast cancer [69].

Based on these previous reports, we also evaluated the effects of endocan knockdown on ERK 1/2 activation in A549 cells. As shown, the degree of ERK 1/2 phosphorylation was significantly reduced in cells where endocan expression was blocked. 

As reported, AKT and ERK 1/2 are both deeply involved in the regulation of a plethora of cell functions, including cell migration and proliferation [42]. Therefore, we evaluated if endocan knockdown affected A549 cell migration and proliferation. We found a significant reduction in cell migration and proliferation in endocan knockdown cells compared to control cells. Notably, these effects were correlated with a reduction in AKT and ERK1/2 activation. Taken together, these results clearly suggest that endocan effects on cell migration and proliferation can be mediated in an AKT and ERK1/2-dependent manner.

In conclusion, our data expand the knowledge of endocan biology in NSCLC, showing that this PG can regulate the expression of tumor-related genes, including epigenetic regulators such as lncRNAs H19 and HULC; furthermore, it promotes tumor cell migration and proliferation.

## 4. Materials and Methods

### 4.1. Cell Cultures

We used A549 cells which are widely used as an in vitro model for non-small-cell lung carcinoma (NSCLC), characterized by a higher endocan expression compared to healthy lungs [70]. 

A549 cells were obtained from ATCC (Manassas, VA, USA) and cultured in 75 cm^2^ plastic flasks containing RPMI-1640 medium (Thermo Fisher Scientific, Milano, Italy). Cells were supplemented with 10% fetal bovine serum (FBS), a mixture of antibiotics (streptomycin/penicillin), and incubated in a 5% CO_2_ humidified incubator at 37 °C. Experiments were performed using A549 cells between the third and tenth passages. The culture medium was renewed every 2–3 days.

### 4.2. Cell Transfection

A549 cells were cultured in six-well culture plates at a density of 2.5 × 10^5^ cells/well. Twenty-four hours after plating (time 0), the culture medium was replaced with OPTIMEM (Life Technologies, Carlsbad, CA, USA). Then cells were transfected with an ESM-1 siRNA (endocan siRNA) (50 pmol/well) (Life Technologies, Carlsbad, CA, USA), using the RNAiMAX transfection kit according to the manufacturer’s protocol (Life Technologies, Carlsbad, CA, USA). 

### 4.3. Endocan ELISA Assay

Samples of cell-secreted proteins in the culture medium were collected 24 h after transfection in the presence of 1 nM PMSF and a protease inhibitor cocktail and centrifuged at 10,000× *g* at 4 °C for 10 min. Endocan levels were detected using a commercial ELISA kit (Raybiotech, Peachtree Corners, GA, USA). Briefly, 100 μL of standards and samples were added, respectively, to each well of the coated microplate. 100 μL of biotin-labeled secondary antibodies were then added and incubated for 2.5 h at 37 °C. After the incubation, the liquid was discarded, the plate was washed four times, and then 100 μL of Streptavidin-HRP was added. After an incubation step of 45 min at 37 °C, the liquid was discarded, the plate was washed four times, and 100 μL of chromogenic substrate were added. After an incubation of 30 min at 37 °C, 50 μL of stop solution was added, and the O.D. absorbance of each well was immediately read at λ 450 nm. Endocan values are expressed as pg/n.cells. 

### 4.4. RNA Isolation, cDNA Synthesis, and Real-Time Quantitative PCR Amplification

Total RNA was isolated from A549 cells for real-time quantitative PCR evaluation (qPCR) of endocan, VEGF-A, VEGFR-2, HIF-1α, lncRNAs H19, and HULC expression (mod 7500, Applied Biosystems Inc., Carlsbad, CA, USA) using the TRIzol reagent kit (Thermo Fisher Scientific, Milano, Italy). The first strand of cDNA was synthesized starting from 5.0 μg of total RNA using a high-capacity cDNA archive kit (Applied Biosystems Inc, Carlsbad, CA, USA). Actin-ß and GAPDH were used as endogenous controls to allow relative quantification. The amplified PCR products were quantified by measuring the calculated cycle thresholds (Ct) of endocan, VEGF-A, VEGFR-2, HIF-1α, lncRNAs H19, HULC, actin-ß, and GAPDH. In addition, a melting curve analysis was always performed to verify the specificity of the reactions. After normalization, the mean value of untreated A549 target levels (CTRL) was chosen as the calibrator, and the results were expressed using the 2^−ΔΔCT^ method and expressed as fold change relative to normal controls. 

### 4.5. Protein Determination

The amount of total protein was determined using the Bio-Rad protein assay system (Bio-Rad, Hercules, CA, USA), using bovine serum albumin (BSA) to obtain a standard curve. 

### 4.6. Protein Extraction and Western Blot Analysis 

Protein extraction from cells was performed using a Cell Extraction Buffer Kit (Life Technologies, Carlsbad, CA, USA) containing a protease inhibitor cocktail (Sigma-Aldrich, Mailand, Italy), 1 nM phenylmethylsulfonyl fluoride (PMSF), and a phosphatase inhibitor cocktail (Sigma-Aldrich, Mailand, Italy). 20 µg of the protein extracted sample was mixed with Laemmli sample buffer with β-mercaptoethanol and separated on SDS-polyacrylamide gels. β-actin protein was used as an endogenous control. Protein samples were blotted on PVDF membranes (Amersham Bioscience, Buckinghamshire, UK) using a specific transfer buffer. After washing in TBS 0.1% Tween 20 buffer and incubation with 2% BSA blotting buffer, membranes were incubated with specific primary antibodies for endocan (Abnova, Taipei, Taiwan), the phosphorylated and total forms of both ERK 1/2 and AKT (Cell Signaling, Danvers, MA, USA), overnight at 4 °C. The next day, the blots were washed in three stages in wash buffer (TBS 0.1% Tween 20) and incubated with the diluted (1:5000) secondary polyclonal antibody (goat anti-rabbit conjugated with peroxidase, purchased at Abcam (Cambridge, UK) in TBS 0.15% Tween 20 buffer. After 1 h of gentle shaking, the blots were washed five times in a wash buffer, and images were achieved and quantified by scanning densitometry using a bio-image analysis system (C-DiGit, Li-cor, Lincoln, NE, USA). 

### 4.7. In Vitro Scratch Assay on A549 Cells 

The scratch assay was performed to evaluate the effects of endocan knockdown on cell migration. A549 cells were grown on 24-well plates at a density of 7.0 × 10^4^. Then, cells were transfected with the endocan siRNA (12 pmol/well) and incubated for an additional 24 h. A linear scratch was created on the cell monolayer using a 20 μL sterile pipette tip. Cells were carefully washed with phosphate-buffered saline (PBS) to remove any scratched cells and further incubated at 37 °C in reduced serum RPMI (0.2% FBS). Relative migration of cells was estimated by taking representative images of the scratch area after 0 h, 3 h, 6 h, and 12 h using a microscopic graduated scale (μm). 

The percentage (%) of wound closure was calculated according to the following algorithm:

$$Wound\;Closure\;\%=\left[\frac{A_{t=0}-A_{t=∆t}}{A_{t=0}}\right]\;\times \;100\%$$

*A*_*t* = 0_ corresponds to the initial wound area, and *A*_*t* = Δ*t*_ corresponds to the wound area after n hours of the initial scratch. Values were expressed as μm^2,^ and images were analyzed using the Image J software [71]. Experiments were performed in triplicate, and untransfected cells were used as controls. 

### 4.8. Cell Proliferation Assay (MTT)

MTT assay was performed to evaluate the effects of endocan silencing on cell proliferation. A549 cells were seeded in 24-well plates at a density of 5.0 × 10^4^ 24 h before treatment with the endocan siRNA. The culture medium was replaced with fresh RPMI (10% FBS) without antibiotics 6 h after transfection, and cells were incubated for 12 h, 24 h, 48 h, and 72 h. Then 400 μL of a mixture constituted by the tetrazolium dye MTT 3-(4,5-dimethylthiazol-2-yl)-2,5-diphenyltetrazolium bromide (Sigma Aldrich, Milan, Italy), dissolved in sterile filtered PBS (5 mg/mL), was added into each well 3 h before the end of the incubation. Then the medium was removed, the insoluble formazan crystals were dissolved in dimethyl sulfoxide (DMSO 1 mL/well) addition, and the OD was read at λ 540 and λ 670 nm. The results were expressed as a percentage (%) increase with respect to control at T0 and reported as means ± standard deviation (S.D.).

### 4.9. Statistical Analysis 

All data are expressed as mean ± standard deviation (S.D.). Statistical significance was calculated with the Student’s *t*-test for unpaired data, and the statistical significance of differences was set at *p* less than 0.05. For the scratch migration assays and the proliferation assay, a two-way ANOVA followed by Tukey’s post hoc test and multiple *t*-tests were performed. All statistical tests were carried out using the software GraphPad Prism (version 7.04 for Windows). All experiments were repeated at least three times (figure legends report the exact number of replicates of each experiment) to ensure reproducibility.

## Data Availability

The data presented in this study are available on request from the corresponding author.

## Supplementary Materials

The following supporting information can be downloaded at: https://www.mdpi.com/article/10.3390/ijms24098178/s1.
