# Supplementary material for: Endocan Promotes Pro-Tumorigenic Signaling in Lung Cancer Cells: Modulation of Cell Proliferation, Migration and lncRNAs H19 and HULC Expression
Source: Int J Mol Sci. 2023 May 3;24(9):8178. doi: 10.3390/ijms24098178 (PMC10179037; doi:10.3390/ijms24098178)
Supplement: Supplementary file 1 [file ijms-24-08178-s001.zip › ijms-2313450-supplementary.pdf]

Caspase-3 protein assay

Caspase-3 activity was evaluated as a marker of apoptosis activation using a Caspase-3/CPP32 Colorimetric Protease Assay Kit (Thermo Fisher Scientific, Milano, Italy), according to manufacturer instructions. Briefly, 24 h after transfection, $1x10^6$ cells for each sample were collected and re-suspended in the chilled Cell Lysis Buffer for 10 min. After centrifugation for 1 minute at 10.000 g, the supernatant was transferred to a new tube and put on ice. After protein quantification, 50 µg of proteins were added to the assay mixture, containing DTT and the caspase-3 chromophore substrate DEVD-pNA, and incubated for 2 h at 37 °C. To provide a positive control, apoptosis was induced by treating A549 cells with staurosporine 1µM. Specific inhibition of caspase-3 was provided by the addition of caspase inhibitor Z-DEVD-FMK. The absorbance was measured using a spectrophotometric microplate reader set at λ 405 nm (Das srl Italy). Values were expressed as relative optical density (OD).

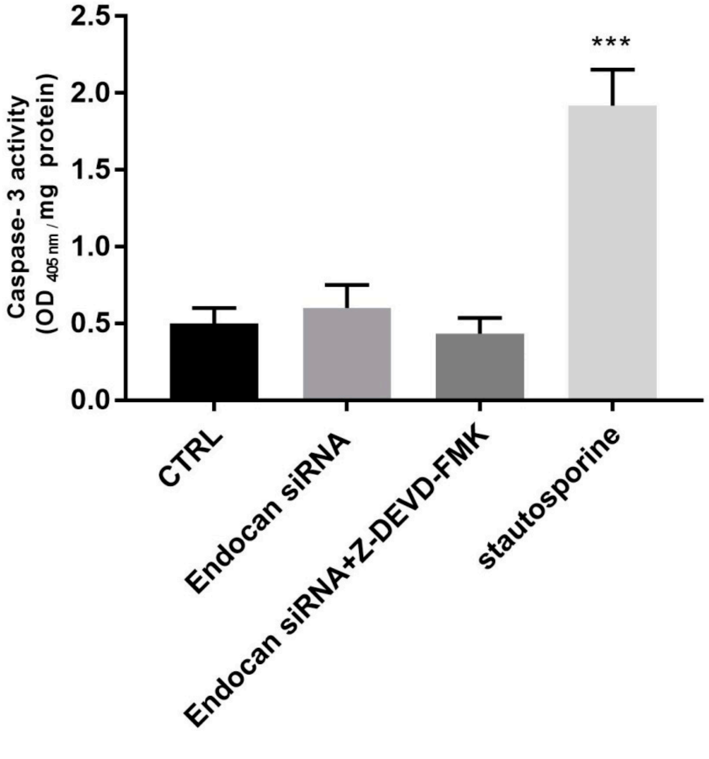

Figure S1. Caspase-3 activity in A549 treated with endocan siRNA , Z-DEVD-FMK or Staurosporine and control cells. Values are mean ± SD of three experiments and are expressed as optical density (OD) at 405 nm /mg protein. ***p<0.001 vs Control
